# Draft Genome Sequence of *Salmonella enterica* subsp. *enterica* Serotype Typhimurium Sequence Type 313, Isolated from India

**DOI:** 10.1128/MRA.00990-18

**Published:** 2018-08-30

**Authors:** Sohan Rodney Bangera, Shashikiran Umakanth, Asish K. Mukhopadhyay, Pimlapas Leekitcharoenphon, Goutam Chowdhury, Rene S. Hendriksen, Mamatha Ballal

**Affiliations:** a Enteric Diseases Division, Department of Microbiology, Kasturba Medical College, Manipal Academy of Higher Education, Manipal, India; b Department of Medicine, Dr. TMA Pai Hospital, Manipal Academy of Higher Education, Udupi, India; c Division of Bacteriology, National Institute of Cholera and Enteric Diseases, Kolkata, India; d Research Group for Genomic Epidemiology, National Food Institute, Technical University of Denmark, Kongens Lyngby, Denmark; Georgia Institute of Technology

## Abstract

Salmonella enterica subsp. enterica serotype Typhimurium sequence type 313 (ST313) is most commonly associated with invasive nontyphoidal Salmonella disease in Africa among patients with HIV infection and malignancy. Here, we report a draft genome sequence of S. Typhimurium ST313, isolated from an elderly immunosuppressed patient from India with non-Hodgkins lymphoma.

## Abstract (precis)

Salmonella enterica subsp. enterica serotype Typhimurium sequence type 313 (ST313) is most commonly associated with invasive nontyphoidal Salmonella disease in Africa among patients with HIV infection and malignancy.

## ANNOUNCEMENT

Salmonella enterica subsp. enterica serotype Typhimurium sequence type 313 (ST313) is a novel sequence type identified solely in patients in sub-Saharan Africa with an invasive disease often associated with advanced HIV infection and malignancy (1). A stool specimen from a patient with mantle cell lymphoma was cultured on Hekton enteric agar following overnight incubation at 37°C, and a nontyphoidal Salmonella sp. was identified following the standard protocol (2). The culture was then subjected to matrix-assisted laser desorption ionization–time of flight mass spectrometry (MALDI-TOF MS) and serotyped at the National Institute of Cholera and Enteric Diseases (NICED) (Kolkata, India), identifying it as S. Typhimurium. DNA was extracted and subjected to PCR for the detection of virulence genes, which showed positive results for the genes *invA*, *spvC*, *sopB*, and *stn*. The extracted DNA was consigned to the Technical University of Denmark for whole-genome sequencing (WGS) and further analysis.

The genomic DNA was extracted with an Invitrogen Easy-DNA kit. The concentration of the DNA was marked using a Qubit double-stranded DNA (dsDNA) broad-range (BR) assay kit. The genomic DNA was formulated for Illumina paired-end sequencing utilizing the Illumina Nextera XT1 guide (number 150319425031942) following protocol revision C. Fragments of pooled Nextera XT libraries were charged onto an Illumina MiSeq reagent cartridge by using a MiSeq reagent kit (version 2) and 500 cycles with a standard flow cell. The libraries were sequenced by applying the Illumina MiSeq platform and MiSeq Control Software version 2.3.0.3. The strain was paired-end sequenced. The raw reads were assembled using the Assemble pipeline (version 1.0), accessible from the Center for Genomic Epidemiology (CGE) (http://cge.cbs.dtu.dk/services/all.php) on the basis of Velvet algorithms for *de novo* short read assembly (3). The genome is a draft version with 79 contigs, and the genome size is 4,926,711 bp with 52.1% G+C content. It contains 4,654 genes.

**FIG 1:**
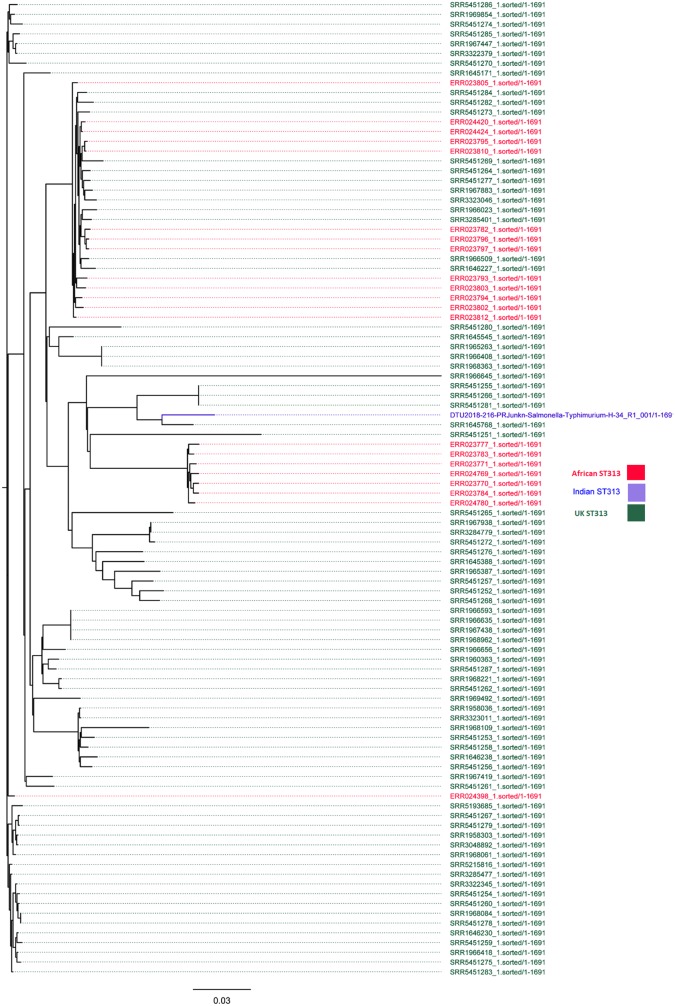
Phylogenetic tree of *Salmonella* Typhimurium ST313 strains isolated from the United Kingdom, Africa, and India. CSI Phylogeny 1.4 (Call SNPs & Infer Phylogeny) (https://cge.cbs.dtu.dk/services/CSIPhylogeny/) was used to build the single-nucleotide polymorphism (SNP) tree. CSI Phylogeny calls SNPs, filters the SNPs, does site validation, and infers a phylogeny based on the concatenated alignment of the high-quality SNPs. The FigTree tree figure drawing tool (version 1.4.3) (2006–2016) (http://tree.bio.ed.ac.uk/software/figtree/) from the Andrew Rambaut Institute of Evolutionary Biology, University of Edinburgh, was used to display the phylogenetic tree.

The assembled sequences were analyzed using bioinformatics tools available from CGE. This process included identifying the ST using the multilocus sequence typing (MLST) tool (version 1.7) (4), which detected the sequence type as ST313. The ResFinder 3.0 tool (5) detected no antimicrobial resistance genes. The genomes of previously described African and UK ST313 isolates were downloaded from the ENA database and compared by WGS-based single-nucleotide polymorphism analysis with the Indian ST313 isolate using CSI phylogeny (version 1.4) (6) to investigate phylogenetic relationships (Fig. 1). The phylogenetic tree analysis demonstrates that the sequence most closely related to that of the Indian ST313 isolate was the UK ST313 isolate (Sequence Read Archive number SRR1645768). Unlike the African ST313 isolate, the Indian ST313 isolate was widely associated with acute gastroenteritis and was susceptible to antimicrobial agents (7). There was a lack of association between the isolate source and travel to the United Kingdom or Africa in this present case. Discovery of a single strain of ST313 in India that is responsible for acute gastroenteritis and positive for invasive genes reveals a previously unknown diversity in ST313. This study in a developing country like India highlights the importance of the widespread introduction of WGS technology, which enables understanding of the epidemiology and microbiology of infectious diseases.

### Data availability.

This whole-genome sequencing project has been deposited in the NCBI Sequence Read Archive under the accession number ERR2676743 (BioProject number PRJEB27562). According to a PubMed search, this is the first time that ST313 has been found outside sub-Saharan Africa and was not associated with any reports of travel to the African continent.

## References

[1] Ashton PM, Owen SV, Kaindama L, Rowe WPM, Lane CR, Larkin L, Nair S, Jenkins C, de Pinna EM, Feasey NA, Hinton JCD, Dallman TJ 2017 Public health surveillance in the UK revolutionizes our understanding of the invasive *Salmonella* Typhimurium epidemic in Africa. Genome Med 9:92. doi:10.1186/s13073-017-0480-7. 29084588 PMC5663059

[2] Winn WC Jr, Allen SD, Janda WM, Koneman E, Procop G, Schreckenberger PC, Woods GL 2006 Koneman’s colour atlas and textbook of diagnostic microbiology, 6th ed, p 93–107. Lippincott, Philadelphia, PA.

[3] Zerbino DR, Birney E 2008 Velvet: algorithms for de novo short read assembly using de Bruijn graphs. Genome Res 18:821–829. doi:10.1101/gr.074492.107. 18349386 PMC2336801

[4] Larsen MV, Cosentino S, Rasmussen S, Friis C, Hasman H, Marvig RL, Jelsbak L, Sicheritz-Pontén T, Ussery DW, Aarestrup FM, Lund O 2012 Multilocus sequence typing of total-genome-sequenced bacteria. J Clin Microbiol 50:1355–1361. doi:10.1128/JCM.06094-11. 22238442 PMC3318499

[5] Zankari E, Hasman H, Kaas RS, Seyfarth AM, Agersø Y, Lund O, Larsen MV, Aarestrup FM 2013 Genotyping using whole-genome sequencing is a realistic alternative to surveillance based on phenotypic antimicrobial susceptibility testing. J Antimicrob Chemother 68:771–777. doi:10.1093/jac/dks496. 23233485

[6] Kaas RS, Leekitcharoenphon P, Aarestrup FM, Lund O 2014 Solving the problem of comparing whole bacterial genomes across different sequencing platforms. PLoS One 9:e104984. doi:10.1371/journal.pone.0104984. 25110940 PMC4128722

[7] Ramachandran G, Panda A, Higginson EE, Ateh E, Lipsky MM, Sen S, Matson CA, Permala-Booth J, DeTolla LJ, Tennant SM 2017 Virulence of invasive *Salmonella* Typhimurium ST313 in animal models of infection. PLoS Negl Trop Dis 11:e0005697. doi:10.1371/journal.pntd.0005697. 28783750 PMC5559095

